# Growth and aging: a common molecular mechanism

**DOI:** 10.18632/aging.100040

**Published:** 2009-04-20

**Authors:** Mikhail V. Blagosklonny, Michael N. Hall

**Affiliations:** ^1^Roswell Park Cancer Institute, Buffalo, NY 14263, USA; ^2^Biozentrum, University of Basel, CH4056 Basel, Switzerland

**Keywords:** aging, growth, rapamycin, TOR, mitochondria

## Abstract

It is commonly assumed that growth and aging are somehow linked, but the nature of this link has been elusive. Here we review the aging process as a continuation of TOR-driven growth. TOR is absolutely essential for developmental growth, but upon completion of development it causes aging and age-related diseases. Thus, the nutrient-sensing and growth-promoting TOR signaling pathway may provide a molecular link between growth and aging that is universal from yeast to human.

## Introduction

At first glance, growth and aging appear to be opposites. Growth is energy-driven synthesis of macromolecules from simple nutrients, an increase of order and a decrease of entropy. Aging is decay, a loss of order and a rise of entropy. Seemingly, growth and aging are mutually exclusive. Forever proliferating cells, such as legendary hydras, do not show signs of aging. In contrast, when an organism ceases to grow, aging follows. However, manipulations that decrease growth also decrease aging and prolong life span. For example, calorie restriction (reduced nutrient intake) reduces growth and increases longevity in diverse species from yeast to mice. Rapamycin, which inhibits growth in yeast, decelerates yeast aging. Inactivation of the growth-promoting insulin/IGF-1 signaling pathway increases life span, from worms to mice. Why do growth-inhibiting conditions slow down aging? Are growth and aging mechanistically similar? As we discuss here, growth and aging may not be opposites but rather a continuation of one another driven by the same molecular pathway. Aging and growth may be linked in a way that growth produces aging. In other words, excessive growth is a driving force for aging. The molecular pathway that drives both growth and aging appears to be the evolutionarily conserved TOR (target of rapamycin) pathway.

### The TOR pathway

TOR (Target Of Rapamycin), as its name indicates, was originally discovered, in yeast, as the target of the antifungal drug rapamycin. Rapamycin is a natural secondary metabolite produced by soil bacteria to inhibit growth of fungal competitors. Thus, it is a mirror image of penicillin that is produced by fungi to inhibit bacterial growth. Remarkably, TOR is structurally and functionally conserved from yeast to human (including worms, flies, plants and mice) as an essential, central controller of cell growth [1]. TOR in mammals (mTOR) controls cell growth and metabolism in response to nutrients (e.g., amino acids), growth factors (e.g., insulin, IGF-1, PDGF), and cellular energy status (ATP). Nutrients are the dominant TOR input as high levels of amino acids can compensate for an absence of the other mTOR inputs but not vice versa [2], and only nutrients activate TOR in unicellular organisms. The growth factor signaling pathway, grafted onto the more ancestral nutrient sensitive TOR pathway, co-evolved with multicellularity. TOR activates cell growth by positively and negatively regulating several anabolic and catabolic processes, respectively, that collectively determine mass accumulation. The anabolic processes include transcription, protein synthesis, ribosome biogenesis, nutrient transport, and mitochondrial metabolism. Conversely, TOR negatively regulates catabolic processes such as mRNA degradation, ubiquitin-dependent proteolysis, and autophagy. TOR is an atypical serine/threonine kinase that is found in two functionally and structurally distinct multiprotein complexes, TORC1 and TORC2 (mTORC1 and mTORC2 in mammals), each of which signals via a different set of effector pathways. TORC1 is rapamycin sensitive whereas TORC2 is rapamycin insensitive. The best-characterized phosphorylation substrates of mTOR are S6K and 4E-BP1 via which mTORC1 controls translation, and Akt/PKB via which mTORC2 controls cell survival and other processes [3]. Like TOR itself, the two TOR complexes and the overall architecture of the TOR signaling network appear to be conserved from yeast to human [1,4]. TOR and many of the processes it controls have also been shown to play a role in aging (in addition to growth) in a wide variety of organisms, as described below.

### Aging in budding yeast

Budding yeast *S. cerevisiae* is a particularly useful model system to study aging because it can be used to study both replicative aging and chronological aging. Replicative aging is measured by the number of daughter cells (divisions) a mother cell can produce. Chronological aging, also referred to as postmitotic aging, is measured by the length of time a non-dividing cell can survive. Inhibition of TORC1 signaling in yeast extends both replicative [5] and chronological [6] life span. Thus, TOR appears to promote aging regardless of physiological context (mitotic or post-mitotic cells).

In yeast, the link between growth and aging has been known sinse the 1950's is particularly spectacular. Yeast cells grow larger as they grow older [7,8]**. **The yeast *S. cerevisiae* typically divides asymmetrically to give a large mother cell and a smaller daughter cell. As mother cells become old, they enlarge and produce daughter cells that are larger than daughters derived from young mother cells. Like large mothers, large daughter cells have shorter replicative life span [8].

The use of unicellular yeast to study aging is revealing also because here the cell is the organism. Therefore, it is a model for cell aging and organismal longevity. As we discuss below, TOR also controls longevity in multicellular organisms.

### TOR and aging from worm to mammals

Inhibition of TOR signaling enhances life span in worms, flies and possibly mammals. The nematode *C. elegans* contains a constant number of post-mitotic cells and lives about twenty days. The first demonstration that TOR controls life span was by Vellai et al [9] who showed that knocking down TOR in *-C. elegans* more than doubles this worm's normal life span. They examined specifically the role of TOR in aging based on the prior knowledge that both TOR and life span are regulated by nutrients and insulin. Indeed, a large body of earlier, groundbreaking work showing that calorie restriction [10,11] or down regulation of insulin/IGF-1 signaling [12,13] extends life span is consistent with the observation that down regulation of TOR also extends life span. Interestingly, inhibition of TOR starting either during development or on the first day of adulthood gives comparable life span extension, suggesting that TOR controls longevity mainly, if not exclusively, during adulthood [9]. Jia et al. [14] subsequently demonstrated that worms deficient in raptor, a TORC1-specific subunit, also have an extended life span, indicating that TOR promotes aging via at least TORC1, if not via both TORCs.

 In *Drosophila*, TOR is required for growth during larval development, and for increases in cellular growth caused by growth factor signaling and nutrient availability [15]. Genetic inhibition of the *Drosophila* TOR pathway, either upstream or downstream of TOR, extends life span [16,17]. Furthermore, reducing the function of *Drosophila* TOR results in decreased lipid stores and glucose levels, and prevents metabolic syndrome [18]. The life span extension is observed upon down regulation of TOR signaling in the fat body [17], underscoring the importance of fat in aging [19-21,12]. Downregulating TOR signaling in the fat body not only extends the life of the fly, it also reduces the size of the entire organism [22], providing yet another link between growth and aging. The apparently central role of TOR signaling in the fly fat body in regulating life span may be recapitulated by mammalian TOR in adipose tissue. In mice, decreased insulin/IGF-1 signaling in adipose tissue, and consequently less active downstream mTOR signaling, increases life span [23,20]. Furthermore, adult-onset growth hormone or IGF-1 deficiency increases life span in rodents [24]. Also, mice deficient for mTORC1 or the mTORC1 effector S6K are protected against age- and diet-induced obesity [25,26]. Rapamycin is currently being tested for its ability to extend lifespan in mice in the National Interventions Testing Program.

Finally, calorie restriction, in which nutrient intake is restricted to 60-70% that of voluntary levels, increases life span in most species including mammals [10-12]. Although anti-aging mechanisms of CR are still disputed, one of the mechanisms is likely inactivation of the TOR pathway. Indeed, taking into account that i) inhibition of TOR extends life span and ii) nutrients activate TOR, the mechanism of how restriction of nutrients can increase life span seems apparent.

### Downstream of TOR

How does TOR promote aging in response to nutrients? In other words, which of the many TORC1-controlled processes that are either up regulated or down regulated upon nutrient deprivation (TORC1 inactivation) leads to longer life? Recent evidence suggests that TORC1 controls aging via several of its downstream processes, including autophagy, ribosome biogenesis and protein synthesis, transcription, and mitochondrial activity. Indeed, there is a remarkable correlation between TOR-controlled processes and processes in aging. It is also important to note that these processes constitute the normal program via which TOR controls cell growth, suggesting that TOR control of aging is an extension or continuation of its control of growth.

TORC1 inhibits autophagy, a process of bulk degradation of proteins and organelles by lysosomes [27]. Autophagy is inhibited in aging and age-related diseases [28]. Restoration of autophagy depletes mitochondria with deleterious mtDNA mutations but spares their normal counterparts [29]. Furthermore, autophagy is essential for life span extension at least in worms [30]. This suggests that TORC1 promotes aging in part via inhibition of autophagy.

 TORC1 activates ribosome biogenesis and protein synthesis. Recent studies show that inhibition of ribosome biogenesis and global protein synthesis extends life span [31-34]. Reducing the levels of ribosomal proteins and translation initiation factors extends life span in both yeast and worms. Thus, this is consistent with the notion that TORC1 may promote aging via activation of ribosome biogenesis and protein synthesis.

TORC1 in yeast negatively regulates the stress-activated transcription factors GIS1 and MSN2/4. Both transcription factors are required for life span extension upon down regulation of TOR [35,36]. A longevity-related gene up regulated by MSN2/4 upon TOR inhibition is the nicotinamidase gene *PCN1*. Interestingly, nicotinamidase converts nicotinamide to NAD^+^ which in turn activates SIR2, suggesting that TOR and sirtuins are part of the same longevity pathway [35]. Furthermore, as discussed below, TOR negatively regulates mitochondrial gene expression to limit life span [37].

TORC1 controls mitochondrial activity, but in different ways depending on the organism. In yeast, TORC1 inhibits mitochondrial respiration whereas in mammals (at least in muscle) it stimulates respiration [37-42]. This divergence in regulation is probably related to the fact that glucose, a nutrient sensed by TORC1, triggers anaerobic fermentation in yeast. A similar glucose-dependent shift in respiration does not occur in mammals. Consistent with the above, increased mitochondrial respiration extends life span in yeast whereas in mammalian cells life span extension correlates with reduced respiration [37,39,43]. However, the role of mitochondria in life span extension remains elusive, particularly with the recent demonstration that TORC1 in mammalian adipose tissue, like in yeast, negatively controls respiration [26].

Accumulation of aggregation-prone proteins is involved in neurodegeneration. TOR causes neurodegeneration in a *Drosophila* tauopathy model [44]. The TOR pathway is involved in Alzheimer's disease by increasing Tau protein synthesis [45]. Furthermore, rapamycin enhances clearance of pathologic proteins and thereby reduces their toxicity [46].

As we discuss below, overactive TOR seems to be involved in the hypertrophic phenotype of aging mammalian cells, thus linking TOR mediated cell hypertrophy to organismal aging. In contrast, a replicative limit has never been shown to be important *in vivo* [47]. It is a hyperthropic, secretory phenotype of aging cells that can be linked to organismal aging [48-50].

### Hypertrophic phenotype of aging cells

If growth and aging are mechanistically linked, are older cells larger? In yeast, old cells are large and cell size predicts replicative life span [51,52]. This also appears to be the case for senescent mammalian cells. An increase in cell size is a hallmark of senescent fibroblasts [53]. Their cell volume is several fold greater compared with proliferating cells. Cell size is progressively increased in cell culture as cells progress toward senescence [54-56]. Furthermore, it was suggested 20 years ago that cell size is a marker of cell senescence [54,57]. Ironically, TOR had not been discovered at that time and the significance of this phenomenological observation was unclear. The notion that TOR is involved in both growth and aging now provides a mechanistic explanation for an old observation.

Cell growth is an increase in cell volume, or mass, due to metabolic activity including synthesis of macromolecules (RNA, protein, lipid) and organelles. If a cell grows without division, it becomes hypertrophic. In other words, when the cell cycle is blocked in the presence of growth-promoting signaling, then cells increase in size [56,58,59].

Thus, cell growth is counterbalanced by cell division such that cells maintain a characteristic size. The simplest way to cause both cell hypertrophy and cell senescence is to prevent cell division without inhibiting cell growth. Inhibition of mTOR with rapamycin decreases the hypertrophic cell phenotype caused by induction of the CDK inhibitor p21 [58-60].

All these observations suggest that mTOR signaling plays a role in aging of single cells. How is this related to aging of multicellular organisms? As discussed elsewhere [1,61], TOR-driven alterations can be linked to metazoan aging and, in particular, diseases of aging such as cancer, metabolic syndrome, atherosclerosis, hypertension and hypertrophic heart.

### Rapamycin in humans

Rapamycin is given to renal transplant patients everyday for several years to prevent organ rejection. We view this as an unintentional clinical trial of a potentially anti-aging drug. First, in such patients, rapamycin unexpectedly turned out to prevent cancer [62-64] and even cured some types of pre-existing tumors [65,66]. Second, 2 years after transplantation, body-mass index was significantly lower in the rapamycin-based treatment arm compared to cyclosporine, indicating that rapamycin prevents obesity [67].

Rapamycin is safe enough to be used in healthy volunteers to study its pharmacokinetics [68-70]. In healthy volunteers, a single dose was not associated with side effects. In 11 healthy men (29 years old, BMI 23 kg/m^2^), 6 mg of rapamycin decreased S6K phosphorylation, preventing insulin resistance caused by nutrients. Thus, the activity state of the mTOR pathway can modulate insulin sensitivity in humans and mTOR inhibitors prevent nutrient-induced insulin resistance [70].

### Why TOR? 

Cell growth and division are the two most fundamental features of life. Using simple compounds and energy, living organisms build macromolecules according to their own plan, transforming non-self to self. Not surprisingly, the growth-controlling TOR signaling pathway is conserved from yeast to human. In unicellular organisms, it maximizes growth as long as nutrients are available. However, life-promoting TOR signaling seems also to contain seeds of death. Aging and its manifestations such as age-related diseases appear with excessive growth-promoting signaling, when actual growth is not longer possible. Aging is not programmed, of course, but is an aimless continuation of the same process that drives developmental growth. Since aging does not limit life span in the wild, switch-off of this ‘growth program' cannot evolve. Growth should be robust and not be slowed down to avoid aging. Furthermore, the aging-growth program cannot be switched off by an accidental mutation, because such a mutation would be lethal or at least reduce fitness during development. Yet, TOR can be inhibited pharmacologically.

## References

[1] Wullschleger S, Loewith R, Hall MN (2006). TOR signaling in growth and metabolism. Cell.

[2] Avruch J, Hara K, Lin Y, Liu M, Long X, Ortiz-Vega S, Yonezawa K (2006). Insulin and amino-acid regulation of mTOR signaling and kinase activity through the Rheb GTPase. Oncogene.

[3] Manning BD, Cantley LC (2007). AKT/PKB signaling: navigating downstream. Cell.

[4] Powers T (2007). TOR signaling and S6 kinase 1: Yeast catches up. Cell Metab.

[5] Kaeberlein M, Powers RW, Steffen KK, Westman EA, Hu D, Dang N, Kerr EO, Kirkland KT, Fields S, Kennedy BK (2005). Regulation of yeast replicative life span by TOR and Sch9 in response to nutrients. Science.

[6] Powers RW, Kaeberlein M, Caldwell SD, Kennedy BK, Fields S (2006). Extension of chronological life span in yeast by decreased TOR pathway signaling. Genes Dev.

[7] Austriaco NRJ (1996). Review: to bud until death: the genetics of ageing in the yeast, Saccharomyces. Yeast.

[8] Kennedy BK, Austriaco NRJ, Guarente L (1994). Daughter cells of Saccharomyces cerevisiae from old mothers display a reduced life span. J Cell Biol.

[9] Vellai T, Takacs-Vellai K, Zhang Y, Kovacs AL, Orosz L, Muller F (2003). Genetics: influence of TOR kinase on lifespan in C. elegans. Nature.

[10] Bordone L, Guarente L (2005). Calorie restriction, SIRT1 and metabolism: understanding longevity. Nat Rev Mol Cell Biol.

[11] Guarente L, Picard F (2005). Calorie restriction - the SIR2 connection. Cell.

[12] Kenyon C (2005). The plasticity of aging: insights from long-lived mutants. Cell.

[13] Kimura KD, Tissenbaum HA, Liu Y, Ruvkun G (1997). daf-2, an insulin receptor-like gene that regulates longevity and diapause in Caenorhabditis elegans. Science.

[14] Jia K, Chen D, Riddle DL (2004). The TOR pathway interacts with the insulin signaling pathway to regulate C. elegans larval development, metabolism and life span. Development.

[15] Zhang H, Stallock JP, Ng JC, Reinhard C, Neufeld TP (2000). Regulation of cellular growth by the Drosophila target of rapamycin dTOR. Genes Dev.

[16] Tatar M, Kopelman A, Epstein D, Tu MP, Yin CM, Garofalo RS (2001). A mutant Drosophila insulin receptor homolog that extends life-span and impairs neuroendocrine function. Science.

[17] Kapahi P, Zid BM, Harper T, Koslover D, Sapin V, Benzer S (2004). Regulation of lifespan in Drosophila by modulation of genes in the TOR signaling pathway. Curr Biol.

[18] Luong N, Davies CR, Wessells RJ, Graham SM, King MT, Veech R, Bodmer R, Oldham SM (2006). Activated FOXO-mediated insulin resistance is blocked by reduction of TOR activity. Cell Metab.

[19] Pinkston JM, Garigan D, Hansen M, Kenyon C (2006). Mutations that increase the life span of C. elegans inhibit tumor growth. Science.

[20] Blüher M, Kahn BB, Kahn CR (2003). Extended longevity in mice lacking the insulin receptor in adipose tissue. Science.

[21] Guarente L, Kenyon C (2000). Genetic pathways that regulate ageing in model organisms. Nature.

[22] Colombani J, Raisin S, Pantalacci S, Radimerski T, Montagne J, Léopold P (2003). A nutrient sensor mechanism controls Drosophila growth. Cell.

[23] Bartke A (2006). Long-lived Klotho mice: new insights into the roles of IGF-1 and insulin in aging. Trends Endocrinol Metab.

[24] Sonntag WE, Carter CS, Ikeno Y, Ekenstedt K, Carlson CS, Loeser RF, Chakrabarty S, Lee S, Bennett C, Ingram R, Moore T, Ramsey M (2005). Adult-onset growth hormone and insulin-like growth factor I deficiency reduces neoplastic disease, modifies age-related pathology, and increases life span. Endocrinology.

[25] Um SH, Frigerio F, Watanabe M, Picard F, Joaquin M, Sticker M, Fumagalli S, Allegrini PR, Kozma SC, Auwerx J, Thomas G (2004). Absence of S6K1 protects against age- and diet-induced obesity while enhancing insulin sensitivity. Nature.

[26] Polak P, Cybulski N, Feige JN, Auwerx J, Rüegg MA, Hall MN (2008). Adipose-specific knockout of raptor results in lean mice with enhanced mitochondrial respiration. Cell Metab.

[27] Klionsky DJ (2007). Autophagy: from phenomenology to molecular understanding in less than a decade. Nat Rev Mol Cell Biol.

[28] Rubinsztein DC (2006). The roles of intracellular protein-degradation pathways in neurodegeneration. Nature.

[29] Gu Y, Wang C, Cohen A (2004). Effect of IGF-1 on the balance between autophagy of dysfunctional mitochondria and apopto-sis. FEBS Lett.

[30] Melendez A, Talloczy Z, Seaman M, Eskelinen EL, Hall DH, Levine B (2003). Autophagy genes are essential for dauer development and life-span extension in C. elegans. Science.

[31] Syntichaki P, Troulinaki K, Tavernarakis N (2007). eIF4E function in somatic cells modulates ageing in Caenorhabditis elegans. Nature.

[32] Pan KZ, Palter JE, Rogers AN, Olsen A, Chen D, Lithgow GJ, Kapahi P (2007). Inhibition of mRNA translation extends lifespan in Caenorhabditis elegans. Aging Cell.

[33] Hansen M, Taubert S, Crawford D, Libina N, Lee SJ, Kenyon C (2007). Lifespan extension by conditions that inhibit translation in Caenorhabditis elegans. Aging Cell.

[34] Steffen KK, MacKay VL, Kerr EO, Tsuchiya M, Hu D, Fox LA, Dang N, Johnston ED, Oakes JA, Tchao BN, Pak DN, Fields S, Kennedy BK, Kaeberlein M (2008). Yeast life span extension by depletion of 60s ribosomal subunits is mediated by Gcn4. Cell.

[35] Medvedik O, Lamming DW, Kim KD, Sinclair DA (2007). MSN2 and MSN4 Link Calorie Restriction and TOR to Sirtuin-Mediated Lifespan Extension in Saccharomyces cerevisiae. PLoS Biol.

[36] Wei M, Fabrizio P, Hu J, Ge H, Cheng C, Li L, Longo VD (2008). Life span extension by calorie restriction depends on Rim15 and transcription factors downstream of Ras/PKA, Tor, and Sch9. PLoS Genet.

[37] Bonawitz ND, Chatenay-Lapointe M, Pan Y, Shadel GS (2007). Reduced TOR signaling extends chronological life span via increased respiration and upregulation of mitochondrial gene expression. Cell Metab.

[38] Schieke SM, Finkel T (2007). TOR and aging: less is more. Cell Metab.

[39] Schieke SM, Phillips D, McCoy JPJ, Aponte AM, Shen RF, Balaban RS, Finkel T (2006). The mammalian target of rapamycin (mTOR) pathway regulates mitochondrial oxygen consumption and oxidative capacity. J Biol Chem.

[40] Cunningham JT, Rodgers JT, Arlow DH, Vazquez F, Mootha VK, Puigserver P (2007). mTOR controls mitochondrial oxidative function through a YY1-PGC-1alpha transcriptional complex. Nature.

[41] Bentzinger CF, Romanino K, Cloëtta D, Lin S, Mascarenhas JB, Oliveri F, Xia J, Casanova E, Costa CF, Brink M, Zorzato F, Hall MN, Rüegg MA (2008). Skeletal muscle-specific ablation of raptor, but not of rictor, causes metabolic changes and results in muscle dystrophy. Cell Metab.

[42] Pan Y, Shadel GS (2009). Extension of chronological life span by reduced TOR signaling requires down-regulation of Sch9p and involves increased mitochondrial OXPHOS complex density. Aging.

[43] Lin SJ, Kaeberlein M, Andalis AA, Sturtz LA, Defossez PA, Culotta VC, Fink GR, Guarente L (2002). Calorie restriction extends Saccharomyces cerevisiae lifespan by increasing respiration. Nature.

[44] Khurana V, Lu Y, Steinhilb ML, Oldham S, Shulman JM, Feany MB (2006). TOR-mediated cell-cycle activation causes neurodegeneration in a Drosophila tauopathy model. Curr Biol.

[45] An WL, Cowburn RF, Li L, Braak H, Alafuzoff I, Iqbal K, Iqbal IG, Winblad B, Pei JJ (2003). Up-regulation of phosphorylated/activated p70 S6 kinase and its relationship to neurofibrillary pathology in Alzheimer's disease. Am J Pathol.

[46] Berger Z, Ravikumar B, Menzies FM, Oroz LG, Underwood BR, Pangalos MN, Schmitt I, Wullner U, Evert BO, O'Kane CJ, Rubinsztein DC (2006). Rapamycin alleviates toxicity of different aggregate-prone proteins. Hum Mol Genet.

[47] Rubin H (2002). The disparity between human cell senescence in vitro and lifelong replication in vivo. Nat Biotechnol.

[48] Campisi J (1998). The role of cellular senescence in skin aging. J Investig Dermatol Symp Proc.

[49] Patil CK, Mian IS, Campisi J (2005). The thorny path linking cellular senescence to organismal aging. Mech Ageing Dev.

[50] Blagosklonny MV (2006). Aging and immortality: quasi-programmed senescence and its pharmacologic inhibition. Cell Cycle.

[51] Zadrag R, Kwolek-Mirek M, Bartosz G, Bilinski T (2006). Relationship between the replicative age and cell volume in Saccharomyces cerevisiae. Acta Biochim Pol.

[52] Bilinski T, Bartosz G (2006). Hypothesis: Cell volume limits cell divisions. Acta Biochim Pol.

[53] Hayflick L (1965). The limited in vitro lifetime of human diploid cell strains. Exp Cell Res.

[54] Angello JC, Pendergrass WR, Norwood TH, Prothero J (1987). Proliferative potential of human fibroblasts: an inverse dependence on cell size. J Cell Physiol.

[55] Sherwood SW, Rush D, Ellsworth JL, Schimke RT (1988). Defining cellular senescence in IMR-90 cells: a flow cytometric analysis. Proc Natl Acad Sci U S A.

[56] Echave P, Conlon IJ, Lloyd AC (2007). Cell size regulation in mammalian cells. Cell Cycle.

[57] Pendergrass W, Angello J, Norwood TH (1989). The relationship between cell size, the activity of DNA polymerase alpha and proliferative activity in human diploid fibroblast-like cell cultures. Exp Gerontol.

[58] Demidenko ZN, Blagosklonny MV (2008). Growth stimulation leads to cellular senescence when the cell cycle is blocked. Cell Cycle.

[59] Demidenko ZN, Blagosklonny MV (2009). Preservation of proliferative potential versus reversal of cellular hypertrophy as a measure of aging-suppression. Aging.

[60] Demidenko ZN, Zubova SG, Bukreeva EI, Pospelov VA, Pospelova TV, Blagosklonny MV (2009). Rapamycin decelerates cellular senescence. Cell Cycle.

[61] Blagosklonny MV (2009). Validation of anti-aging drugs by treating age-related diseases. Aging.

[62] Kauffman HM, Cherikh WS, Cheng Y, Hanto DW, Kahan BD (2005). Maintenance immunosuppression with target-of-rapamycin inhibitors is associated with a reduced incidence of de novo malignancies. Transplantation.

[63] Yakupoglu YK, Buell JF, Woodle S, Kahan BD (2006). Individualization of Immunosuppressive Therapy. III. Sirolimus Associated With a Reduced Incidence of Malignancy. Transplant Proc.

[64] Campistol JM, Eris J, Oberbauer R, Friend P, Hutchison B, Morales JM, Claesson K, Stallone G, Russ G, Rostaing L, Kreis H, Burke JT, Brault Y, Scarola JA, Neylan JF (2006). Sirolimus Therapy after Early Cyclosporine Withdrawal Reduces the Risk for Cancer in Adult Renal Transplantation. J Am Soc Nephrol.

[65] Stallone G, Schena A, Infante B, Di Paolo S, Loverre A, Maggio G, Ranieri E, Gesualdo L, Schena FP, Grandaliano G (2005). Sirolimus for Kaposi's sarcoma in renal-transplant recipients. N Engl J Med.

[66] Pascual J, Fernández AM, Marcén R, Ortuño J (2006). Conversion to everolimus in a patient with arterial hypertension and recurrent cutaneous neoplasia - a case report. Nephrol Dial Transplant.

[67] Rovira J, Marcelo Arellano E, Burke JT, Brault Y, Moya-Rull D, Banón-Maneus E, Ramírez-Bajo MJ, Gutiérrez-Dalmau A, Revuelta I, Quintana LF, Campistol JM, Diekmann F (2008). Effect of mTOR inhibitor on body weight: from an experimental rat model to human transplant patients. Transpl Int.

[68] Leelahavanichkul A, Areepium N, Vadcharavivad S, Praditpornsilpa K, Avihingsanon Y, Karnjanabuchmd T, Eiam-Ong S, Tungsanga K (2005). Pharmacokinetics of sirolimus in Thai healthy volunteers. J Med Assoc Thai.

[69] Leung LY, Lim HK, Abell MW, Zimmerman JJ (2006). Pharmacokinetics and Metabolic Disposition of Sirolimus in Healthy Male Volunteers After a Single Oral Dose. Ther Drug Monit.

[70] Krebs M, Brunmair B, Brehm A, Artwohl M, Szendroedi J, Nowotny P, Roth E, Fürnsinn C, Promintzer M, Anderwald C, Bischof M, Roden M (2007). The Mammalian target of rapamycin pathway regulates nutrient-sensitive glucose uptake in man. Diabetes.

